# Verses

**Published:** 1907-10

**Authors:** 


### VERSES.

### A THANKSGIVING POINTER.

A turkey's age can be told, they say,
  By the teeth—now don't pooh-pooh it—
For it's not by the turkey's teeth—nay, nay!—
But the teeth that try, Thanksgiving Day,
  When the bird is cooked, to chew it.

—Nixon Waterman.

### TOUCHING.

The gentle touch
  Of a pair of lips;
The gentle touch
  Of cool finger-tips;
The gentle touch
  Of a falling leaf;
The gentle touch
  Of a passing grief;
The touch of a joy;
  The touch of pain;
The touch of wind,
  Of the touch of rain;
The touch of time;
  A touch of sin;
The touch of nature
  That makes us kin;
'Most any touch
  Can a mortal brook
That doesn't quite
  Touch his pocketbook.

—Houston Post.

### YOUR WORLD.

The world is neither long nor wide;
'Tis but the little vale
Where you have chosen to abide.
Where you succeed or fail.

The world is but the little sphere
In which you come and go
And are you aiding to its cheer,
Or do you spread its woe?

—Newspaper.

### THE OLD FOLKS.

De ole fokes am er sittin een de chimbly corner dar,
Jess er smokin dere pipes, een er dremin sort er stage,
Tinkin ob de times, way back so far,
Wid brite sunny days ob er pas gon age.

De mos ob dere frens, hab dere lites gon out,
An am lade een de grabe, outen dere site,
But de memory ob dem times wen dey wus yere about,
Am es fresh ter dem now, es de brod daylite.

No times ter dem now, am es good es dem times,
No diffunce how much yer teck em erroun,
So let em erlone, ter dream, or sing himes,
An pray fer ter die, an go een de groun.

Dey am happy no longer, wid dere pains an dere akes,
Dars nuttin ter lib fer, only watin ter die,
So treat em bofe kindly, an for dere sakes,
Wish fer dem hallelooyah, een de sweet by an by.

—B. H. Teague.

